# The choice of treatment and the motivations behind it impact clinical outcomes among patients with adequate control of their rheumatic disease: A real-life study

**DOI:** 10.1371/journal.pone.0315478

**Published:** 2024-12-12

**Authors:** Irazú Contreras-Yáñez, Guillermo A. Guaracha-Basáñez, Diana Padilla-Ortiz, Laura L. Franco-Mejía, Laura V. Vargas-Sánchez, Julia G. Jiménez-Decle, Virginia Pascual-Ramos

**Affiliations:** Department of Immunology and Rheumatology, Instituto Nacional de Ciencias Médicas y Nutrición Salvador-Zubirán (INCMyN-SZ), Mexico City, Mexico; Al Nasiriyah Teaching Hospital, IRAQ

## Abstract

**Introduction:**

Many factors influence how doctors make treatment decisions. The study compares the outcomes of patients with rheumatic diseases and adequate control (AC) whose treating rheumatologists prescribed their first choice of treatment (FCHO) versus the second choice (SCHO) and the motivations behind them. It also investigates the motivations associated with FCHO.

**Patients and methods:**

The study was conducted at an outpatient clinic from February 2023 to February 2024. Patients with an RMD diagnosis were identified using systematic sampling (P-1). After their consultation, their rheumatologists detailed their treatment choice (FCHO vs. SCHO), the motivations behind it, and the outcomes. In a subsample of patients from P-1 and AC (SubP-1), treating rheumatologists repeated the assessment of outcomes at the next scheduled consultation. Descriptive statistics and multivariate regression analysis were used.

**Results:**

There were 703 patients enrolled (P-1), 543 (77.2%) had AC, and 292 (Subp-1) underwent a follow-up evaluation. In P-1 and subP-1, FCHO was prescribed to 644 (91.5%) and 269 (92.1%) patients.

Motivations related to evidence-based medicine and personal experience were more frequently referred to in FCHO. Concerns related to current or future drug shortages and a history of adverse events/intolerance were more frequent in SCHO.

In SubP-1, a higher proportion of patients remained in AC and experienced remission/ improved disease activity with FCHO. Patients who received FCHO experienced a greater risk for favorable outcomes.

The following motivations were associated with FCHO: “It aligns with guidelines”; “solid scientific evidence supporting the treatment effectiveness”; “I am concerned that the shortage of the drug may hinder the continuation of the treatment” and “history of adverse events or intolerance”.

**Conclusions:**

Patients with AC of their underlying RMD, whose rheumatologists prescribed their FCHO, had better outcomes than those who were prescribed SCHO. Evidence-based motivations, rheumatologists´ concern of medication shortage, and patient-related motivations were associated with FCHO.

## Introduction

Rheumatic diseases (RMDs) are a group of complex chronic musculoskeletal disorders. They are mainly characterized by musculoskeletal pain, impaired function, reduced quality of life, common comorbidities, and higher mortality compared to healthy individuals [1–3]. Treatment of patients with RMDs has seen significant progress in recent decades. The available therapeutic options now encompass both traditional and modern medications. Additionally, there are strategic methods involving various agents and non-pharmacologic interventions. These interventions include surgery, physical therapy, and occupational therapy provided by multidisciplinary teams [4]. Finally, advancements in treatment interventions are important. However, they should not overshadow the essential depth and care in the relationship between healthcare providers and individuals with RMDs. This should be our legacy for future generations.

Patient-centered care has been proposed as the best approach for patients with chronic diseases, including RMDs [5]. According to the Institute of Medicine, patient-centered care means "Providing care that is respectful of, and responsive to, individual patient preferences, needs and values, and ensuring that patient values guide all clinical decisions." [6]. This approach necessitates a genuine partnership between individuals and their healthcare providers, where the individual’s needs and aspirations steer healthcare decisions and outcomes [7]. Several barriers have been identified that hinder the implementation of patient-centered care in clinical practice. One significant barrier may be current rheumatologists’ strong emphasis on a perhaps misunderstood concept of evidence-based medicine, which has led to the proliferation and adoption of updated guidelines for the management of patients with RMDs [8–13].

Evidence-based medicine, first defined in 1991 [14], aims to educate front-line clinicians on how to assess the credibility of research evidence. It helps them understand the results of clinical studies and apply those results effectively in their daily practice [15]. At the core of evidence-based medicine is the belief that what is considered reasonable depends on the trustworthiness of the evidence. It also relies on the extent to which we believe that this evidence arises from credible processes [16]. However, evidence-based medicine also recognizes that effective decision-making requires consideration of the environment and context of patients’ values and preferences [15,16]. In spite of this, rheumatologists perceived that clinical practice guidelines might be incompatible with shared decision-making implementation [17].

Several factors affect how doctors make decisions and prescribe treatments. Research indicates that clinical guidelines have different levels of influence on physicians. There is limited evidence regarding the impact of patients on prescribing decisions. Clinicians are increasingly mindful of costs. Furthermore, doctors’ attitudes toward shared decision-making play a significant role as well [18–22]. Meanwhile, the physician gender as a source of implicit bias affecting clinical decision-making has been increasingly recognized [23]. Moreover, patients’ willingness to engage in the decision-making process can vary and change over time. This willingness may depend on factors such as symptom severity, prognosis, race, gender, socioeconomic status, language proficiency, health literacy, and the dynamics of the patient-physician relationship [24–26]. Also, there is growing evidence that emotions play an undeniable role in how patients and physicians work together to make choices, although acknowledgment of that is far from universal [27,28]. Ultimately, the decision-making process leads to a treatment choice that might impact patient outcomes. Further exploration is needed to understand the extent to which treatment choices affect outcomes in patients with RMDs and how clinical contexts shape them.

Taking the above considerations into account, the objective of the study was to compare the clinical outcomes of patients with RMDs who were under control. The study focused on two groups: those whose treating rheumatologists (TRs) prescribed their first choice of treatment (FCHO) and those who received the second choice (SCHO). Additionally, the research aimed to explore the motivations behind the physicians’ treatment decisions, specifically those linked to the FCHO.

## Patients and methods

### Ethics

The Internal Review Board of the Instituto Nacional de Ciencias Médicas y Nutrición Salvador Zubirán (INCMyN-SZ) approved the study with the reference number IRE-4442. Consecutive patients from the outpatient clinic of the Department of Immunology and Rheumatology (OCDIR) were invited to participate in the study. Before enrolling patients, the study was presented to the rheumatologists and trainees in rheumatology assigned to the OCDIR, who agreed to collaborate and provided verbal informed consent. Patients who agreed to participate provided written informed consent.

### Study design, setting, and study population

This prospective study was conducted at the INCMyN-SZ, a tertiary-care level and academic center in Mexico City affiliated with the National Institutes of Health of Mexico, between February, 13^th^ 2023, and February, 15^th^ 2024. Before 2020, patients’ medical coverage was determined based on their socioeconomic status, which was assessed by social workers through interviews and evaluation of their income-to-needs ratio. Patients had to make co-payments for medication, medical services, laboratory tests, and diagnostic imaging studies, depending on their health coverage, which ranged from 0 to 100%. Starting on December 1st, 2020, patients have received full coverage. However, there have been challenges in ensuring an adequate supply of medications. Additionally, full health coverage is provided by various federal institutions, and individuals who are beneficiaries of other health coverage may not be eligible for local benefits.

The OCDIR was staffed with fourteen certified rheumatologists and ten trainees during the study period. Approximately 6,300 patients had at least one clinical assessment, and the total consultations were approximately 18000. Upon their first visit to the OCDIR, patients were assigned a treating rheumatologist, who remained the same throughout the patient’s follow-up unless the patient specifically requested a change. However, patients assigned to trainees would change their rheumatologist upon completion of the trainee’s academic courses**. S1 Table** presents the ten most frequent RMD diagnoses. The OCDIR provides scheduled clinical services four days a week.

From February 2023 to April 2024, patients with an RMD diagnosis based on the treating rheumatologist criteria and a scheduled consultation at the OCDIR (inclusion criteria) were invited to participate. We used systematic sampling to include up to 4–8 patients per day. The total number of patients included during the study period was 708 (Population 1 [P-1]). Subsequently, systematic sampling created a subsample of patients from P-1 (SubP-1) with adequate control of their RMD (AC, see definition below) for prospective evaluations.

### Study maneuvers

All patients who were part of P-1 underwent scheduled medical appointments with their treating rheumatologists. Patients were identified the day before their planned visit to the OCDIR. Those attending the OCDIR were invited to participate on the day of the scheduled visit. Upon agreement, they were instructed to complete the Health Assessment Questionnaire Disability Index (HAQ-DI) [29], the EuroQol- 5 Dimensions (EQ-5D) [30], and the Patient Doctor Relationship Questionnaire-9 items (PDRQ-9) [31]. After each appointment, their assigned rheumatologists were also required to fill out two standardized forms. The first form, “Treatment choice” included their treatment choice (FCHO or SCHO) and all the reasons behind it. The second form (“RMD outcomes”) documented the rheumatologists’ assessment of the current level of disease activity, RMD control, and any changes in treatment [32]. This process was repeated from February 2023 to February 2024 until a sample size of at least 420 medical encounters with different patients was evaluated (please refer to sample size calculation).

Patients who were part of SubP-1 were monitored until their next appointment with their rheumatologists. After the consultation, they were directed to complete the HAQ-DI, EQ-5D, and the PDRQ-9. The treating rheumatologists immediately completed the RMD-outcomes form. This process was repeated until a sample size of at least 289 medical encounters with different patients was completed (please refer to sample size calculation).

In all cases, standardized formats were used.

### Instruments description

#### Treatment choice questionnaire

A literature search failed to identify validated Spanish tools for addressing the primary study objective. Good practice in conducting and reporting survey research was followed [33]. Treatment choice content was proposed by a committee consisting of two rheumatologists attending the OCDIR (VPR, GAGB) and one social worker with a PhD in medical sciences (ICY). The committee agreed on the four components to be included, the items, and their scale responses based on the literature review [18–28]. Then, treatment choice face and content validity were tested by the remaining rheumatologists assigned to the OCDIR. This testing focused on instructions, individual items, the adequacy of the item’s scale response, and the relevance and pertinence of individual items to the survey purpose. The pilot testing included evaluations of 50 medical encounters. Modifications were adopted (**S1 Appendix**).

#### RMD outcomes questionnaire

This tool was created to evaluate the clinical status of the underlying RMD [32]. For this study, RMD outcomes consisted of three categories (please refer to **S2 Table**): the current level of disease activity, RMD control, and treatment recommendations given by the rheumatologist at the end of the consultation. These categories and sub-categories were suggested after at least 80% of the 11 certified rheumatologists affiliated with ORCID reached a consensus (see **S2 Appendix**).

#### HAQ-DI

This index includes 20 items that assess limitations to perform eight activities of daily living: dressing, arising, eating, walking, hygiene, reach, grip, and usual activities. There are two or three questions for each activity/section. The score ranges from 0 to 3, with higher scores translating into more severe disability.

#### EQ-5D

The EQ-5D-3L is a standardized measure of health-related quality of life that evaluates health status in five dimensions: mobility, self-care, usual activities, pain and discomfort, and anxiety and depression. Each dimension has three severity response levels. Respondents must select the statement in each dimension that best describes their health status on the day of the survey. They are then assigned a number (1, 2, or 3) corresponding to the respective severity level: 1 indicates no problems, 2 signifies some problems, and 3 represents extreme problems.

#### PDRQ-9

It is an instrument that evaluates the patient-doctor relationship from the perspective of the patient. Each item of the instrument is a statement about different attributes of the relationship (help, time, trust, understanding, dedication, agreement, availability, contentment, and accessibility), which evaluates the relational and satisfaction aspects. The general score is calculated by the arithmetic mean of the answers of the nine items, from 0 to 5, with higher scores indicating better patient-doctor relationship.

#### Definitions

RMD diagnosis and treating rheumatologist choices (FCHO and SCHO) were based on the physician´s clinical judgment. FCHO was defined as the physician prescription which was ranked as a priority (“the best”) over other treatment options. Emphasis was made that the different cognitive processes behind the clinical judgment (for instance, evidence-based, share making decision, among others) were all valid.

RMD outcomes were based on the attendant rheumatologist criteria. However, pre-specified criteria were suggested for the RMD outcomes categories (**S2 Table**).

We defined *sustained AC* in all the patients from SubP-1 who were in AC at the follow-up evaluation.

We defined a *favorable disease activity course* in all the patients from SubP-1 who were in remission or improved their disease activity level at the follow-up evaluation.

We defined *treatment intensification* when TRs selected the “Treatment was modified because of RMD deterioration/insufficient response” option (See **S2 Table** and **S2 Appendix**).

We defined *normal physical function* when patients had HAQ-DI score ≤0.5 at the follow-up evaluation.

We defined *normal health-related quality of life* as when patients reported no problems with the mobility, self-care, and usual activities dimensions of the EQ-5D-3L and the absence of pain/discomfort and anxiety/depression at the follow-up EQ-5D-3L.

Finally, we defined an *improved patient-doctor relationship* when patients increased their PDRQ-9 scores at the follow-up evaluation.

### Sample size calculation

In order to meet our main goal, we calculated the sample size needed to identify a difference in the percentage of patients who maintained AC during follow-up, comparing medical appointments where the rheumatologist indicated "FCHO" (at 80%) versus "SCHO" (at 70%). In a previous study, we observed that up to 66% of patients in the OCDIR had AC of their RMD [32]. Also, we hypothesized a 10–20% difference in sustained outcomes based on published results observed in specific clinical contexts [34,35]. We used a one-tailed test with a 95% significance level and 80% power. Initially, we had 231 patient-physician encounters with RMD assessment, which was then increased to at least 289 medical encounters, allowing for a 20% loss of non-analyzable data.

Furthermore, we also calculated the sample size to identify a 10–20% prevalence of "SCHO" (arbitrarily hypothesized). In 2023, approximately 6,852 patients in the OCDIR were coded with a definite RMD diagnosis. Ultimately, we required at least 364 medical encounters, with a 95% confidence level and 5% precision.

It’s important to note that the medical encounters analyzed involved different patients.

### Statistical analysis

We conducted descriptive statistics to describe the variables of the patients in groups P-1 and SubP-1. Categorical variables were presented with frequencies and percentages, while continuous variables with normal distribution were described using the mean and standard deviation, and those with non-normal distribution with median and interquartile range (Q25-Q75).

We used appropriate tests to compare variables between the groups FCHO and SCHO. We used the X^2^ test for categorical variables, and for continuous variables with a non-normal distribution, we used the Mann-Whitney U test.

We used hazard ratios (HR) (95% confidence interval [CI]) to estimate the risk of developing favorable outcomes relative to FCHO (comparator SCHO).

We performed multiple logistic regression analyses to identify motivations associated with FCHO as the dependent variable. We constructed a global model and included variables based on their statistical significance in the univariate analysis (p≤0.05). After that, we used a backward selection to define the final model, adjusting for the sex and degree of the treating rheumatologist (certified vs. trainee). Previously, we examined correlations between variables to avoid overfitting the models, but none were relevant (rho≤0.70). The Nagelkerke pseudo-R^2^ test was reported as a measure of model fit goodness. The results were expressed as adjusted Odds Ratios (aOR = exp[*β*]) and their 95% confidence interval (CI). Missing data were below 0.5%, and no imputation was performed.

All statistical analyses were performed using Statistical Package for the Social Sciences version 21.0 (SPSS Chicago IL). A value of p<0.05 was considered statistically significant.

## Results

### Populations description

**Fig 1:**
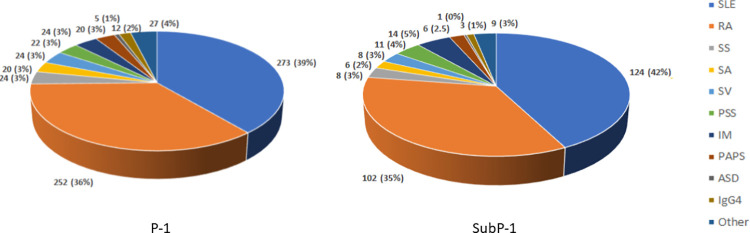
Diagnoses distribution in P-1 and SubP-1. SLE = Systemic Lupus Erythematosus; RA = Rheumatoid Arthritis; SS = Sjogren Syndrome; SS = Scleroderma; SV = Systemic Vasculitis; PSS = Primary Sjogren Syndrome; IM = Inflammatory Myopathies; PAPS = Primary Antiphospholipid Syndrome; ASD = Adult Still Disease; IgG4 = IgG4-related disease.

During the study period, 703 patients were enrolled (P-1), and 543 (77.2%) had AC. Among them, 292 were randomized and underwent a second evaluation of AC of their RMD (SubP-1) after 5 months (4–6). **Table 1** and **Fig 1** summarize their main characteristics and diagnoses. In summary, the patients were mainly middle-aged females with 12 (9–17) years of formal education. The majority had middle-low socioeconomic levels and were living together. Patients had an average of 15 (8–23) years of disease duration; most had AC and some comorbidity. A few had current mental health comorbidity and mentioned at least one hospitalization in the previous year. Regarding patient-reported outcomes measures (PROMs), half of them had some disability. However, the majority perceived no problems with the mobility, self-care, usual activities, and anxiety/depression dimensions of the EQoL-5D.

**Table 1:** Main characteristics of the whole population (P-1) and the subgroup of patients with a follow-up evaluation (SubP-1).

	P-1 N = 703	SubP-1 N = 292
**Socio-demographics**		
Age, years	51 (40–61)	50 (41–60)
Females*	601 (85.5)	256 (87.7)
Years of scholarship	12 (9–16)	12 (9–16)
Living together*	371 (52.8)	158 (54.1)
Formal and non-formal job*	305 (43.4)	123 (42.1)
Access to Social Security benefits*	244 (34.7)	88 (30.1)
Middle-low socioeconomic level*	623 (88.6)	267 (91.4)
Religious beliefs*	638 (90.8)	266 (91.1)
**RMD-related characteristics**		
AC*	543 (77.2)	292 (100)
Disease duration, years	15 (8–23)	15 (8–23)
Comorbidity*	458 (65.1)	185 (63.4)
Current mental health comorbidity*	170 (24.2)	75 (25.7)
Hospitalization in the previous year*	102 (14.5)	41 (14)
Number of hospitalizations/patient^1^	1 (1–1)	1 (1–1)
**Patient-reported outcome measures (PROMs)**		
HAQ-DI score	0.5 (0–1.13)	0.3 (0–0.88)
Patients with disability* (HAQ-DI score ≥0.5)	356 (50.6)	126 (43.2)
Patients with no perceived problems in the EQol-5D* Mobility *Self-care* *Usual activities* *Pain/discomfort* *Anxiety/depression*	489 (69.6) 624 (88.8) 550 (78.2) 299 (45.5) 519 (73.8)	213 (72.9) 266 (91.1) 242 (82.9) 135 (46.2) 217 (74.3)
PDRQ-9 score	5 (4.6–5)	5 (4.6–5)
**RMD-related treatment**		
FCHO*	643 (91.5)	269 (92.1)
SCHO*	60 (8.5)	23 (7.9)
Patients on immunosuppressive drugs*	645 (91.7)	271 (92.8)
Number of immunosuppressive/patient^1^	1 (1–2)	1 (1–2)
Patients on biologics and small molecules*	70 (10)	36 (12.3)
Patient on corticosteroids*	271 (38.5)	109 (37.3)

Data presented as median (IQR) unless

*that shows the number (%) of patients.

^1^Among those with the condition. AC = Adequate control. FCHO = First choice of treatment. SCHO = Second choice of treatment.

SLE and RA were the most frequent RMD diagnoses, and their frequencies represented the highest proportion of RMD diagnoses (**Fig 1**).

### TRs´ treatment choices and motivations behind

In P-1 and SubP-1, FCHO was prescribed to 643 (91.5%) and 269 (92.1%) patients, while SCHO was prescribed to 60 (8.5%) and 23 (7.9%) patients.

**Table 2** compares the reasons behind the treatment choice between physicians who prescribed FCHO and SCHO in P-1. In summary, the motivations related to evidence-based medicine (“It aligns with national and international guidelines” and “There is solid scientific evidence supporting the effectiveness of the treatment”) and personal experience were more frequently observed in FCHO. On the other hand, concerns related to future drug shortages, history of adverse events or intolerance, and current drug shortages were more frequent in SCHO. Similar results were obtained in SubP-1 (please refer to **S3 Table**).

**Table 2:** Comparison of physicians´ motivations behind FCHO and SCHO, in P-1.

	FCHO	SCHO	p
**Physician-related**			
It aligns with national and international guidelines.	639 (99.4)	47 (78.3)	≤0.0001
There is solid scientific evidence supporting the effectiveness of the treatment.	626 (97.4)	47 (78.3)	≤0.0001
I have personal experience with that treatment.	626 (97.4)	55 (91.7)	0.032
I am concerned that the shortage of the drug may hinder the continuation of the treatment for the necessary duration.	125 (19.4)	25 (41.7)	≤0.0001
Other reasons^1^.	19 (3)	4 (6.7)	0.124
**Patient-related**			
Socio-demographics (age, education level, etc. . .).	266 (41.4)	21 (35)	0.410
Relevant comorbidities.	234 (36.4)	23 (38.3)	0.780
History of adverse events or intolerance.	85 (13.2)	23 (38.3)	≤0.0001
Economic motivations: the patient can´t afford the treatment.	152 (23.6)	9 (15)	0.149
Patients´ preference.	221 (34.4)	20 (33.3)	1
Other reasons^2^.	22 (3.4)	4 (6.7)	0.268
**Health-care system related**			
Local shortage	95 (14.8)	17 (28.3)	0.009
National shortage	21 (3.3)	3 (5)	0.450
Patient benefits from local gratuity	346 (53.8)	29 (48.3)	0.421
Patient benefits from social security gratuity	149 (23.2)	15 (25)	0.750
Other reasons^3^.	10 (1.6)	2 (3.3)	0.273

Data presents the number (%) of physicians who selected that motivation. ^1^No reasons were specified in 65.2% of the treatment choice questionnaires with the “Other reasons” option selected. ^2^No reasons were specified in 57.5% of the treatment choice questionnaires with the “Other reasons” option selected, while pregnancy/pregnancy planning/breastfeeding were referred in 19.2%. ^3^No reasons were specified in 94.5% of the treatment choice questionnaires with the Other reasons” option selected.

We also looked at the potential differences in motivations behind treatment choices between certified rheumatologists (445 [63.3%] medical encounters with fourteen physicians) and trainees in rheumatology (258 [36.7%] medical encounters with ten physicians). The results are summarized in **Table 3**. Trainees more often referred to motivations related to evidence-based medicine. In contrast, certified rheumatologists placed greater emphasis on social factors, such as the benefits patients gain from local support. Additionally, they demonstrated a tendency to consider patients’ preferences.

**Table 3:** Comparison of the motivations behind the treatment choice between certified rheumatologists and trainees in rheumatology.

	Clinical encounters with certified rheumatologists, n = 445	Clinical encounters with trainees, n = 258	p
**Physician-related**			
It aligns with national and international guidelines.	430 (96.6)	256 (99.2)	0.039
There is solid scientific evidence supporting the effectiveness of the treatment.	419 (94.2)	254 (98.4)	0.006
I have personal experience with that treatment.	433 (97.3)	248 (96.1)	0.380
I am concerned that the shortage of the drug may hinder the continuation of the treatment for the necessary duration.	103 (23.1)	47 (18.2)	0.128
Other reasons^1^.	10 (22)	13 (5)	0.05
**Patient-related**			
Socio-demographics (age, education level, etc. . .).	188 (48.2)	99 (38.4)	0.340
Relevant comorbidities.	165 (37.1)	92 (35.7)	0.745
History of adverse events or intolerance.	70 (15.7)	38 (14.7)	0.746
Economic motivations: the patient can´t afford the treatment.	104 (23.4)	57 (22.1)	0.711
Patients´ preference.	163 (36.6)	78 (11.1)	0.099
Other reasons^2^.	11 (2.5)	15 (5.8)	0.036
**Health-care system related**			
Local shortage	73 (16.4)	39 (15.1)	0.679
National shortage	11 (2.5)	13 (5)	0.085
Patient benefits from local gratuity	255 (57.3)	120 (46.5)	0.006
Patient benefits from social security gratuity	107 (57.3)	120 (46.5)	0.006
Other reasons^3^.	107 (24)	57 (22.1)	0.580

Data presented as Number (%).

^1^No reasons were specified in 65.2% of the treatment choice questionnaires with the “Other reasons” option selected

^2^No reasons were specified in 57.5% of the treatment choice questionnaires with the “Other reasons” option selected, while pregnancy/pregnancy planning/breastfeeding were referred in 19.2%

^3^No reasons were specified in 94.5% of the treatment choice questionnaires with the Other reasons” option selected.

### Outcomes comparison between medical encounters where patients were indicated rheumatologists FCHO and SCHO

**Table 4** summarizes the key findings. In SubP-1, a higher proportion of patients remained in AC with FCHO compared to SCHO. Additionally, more patients experienced either remission or improved disease activity levels. However, no significant differences in PROMs were observed between FCHO and SCHO. These results were consistent with the risks observed in patients’ outcomes following medical encounters with FCHO and SCHO.

**Table 4:** Comparison of patient outcomes between FCHO and SCHO medical encounters and the relative risks of favorable outcomes associated with FCHO.

	FCHO^1^	SCHO^1^	p	HR, 95% CI, p^2^
Sustained AC	239 (88.8)	16 (69.6)	**0.016**	3.485, 1.327–9.157, **0.011**
Favorable disease activity course	254 (94.4)	19 (82.6)	**0.051**	3.565, 1.077–11.805, **0.037**
Treatment intensification	39 (14.8)	5 (22.7)	0.354	0.597, 0.208–1.713, 0.338
Normal physical function	171 (63.6)	12 (52.2)	0.369	1.599, 0.680–3.761, 0.282
Normal mobility	154 (57.2)	16 (69.6)	0.279	0.586, 0.233–1.471, 0.255
Normal self-care	176 (65.4)	18 (78.3)	0.255	0.526, 0.189–1.461, 0.218
Normal usual activities	168 (62.5)	19 (82.6)	0.069	0.350, 0.116–1.058, 0.063
Absence of pain and discomfort	105 (39)	9 (39.1)	1	0.996, 0.416–2.383, 0.993
Absence anxiety and depression	150 (55.8)	17 (73.9)	0.124	0.445, 0.170–1.163, 0.099
Improved patient-doctor relationship	73 (27.3)	6 (26.1)	1	1.066, 0.405–2.809, 0.897

^1^Data is presented as the number (%) of patients.

^2^HR, 95% CI and p value for relative risk of favorable outcomes for FCHO (comparator SCHO).

### Motivations associated with FCHO

**Fig 2:**
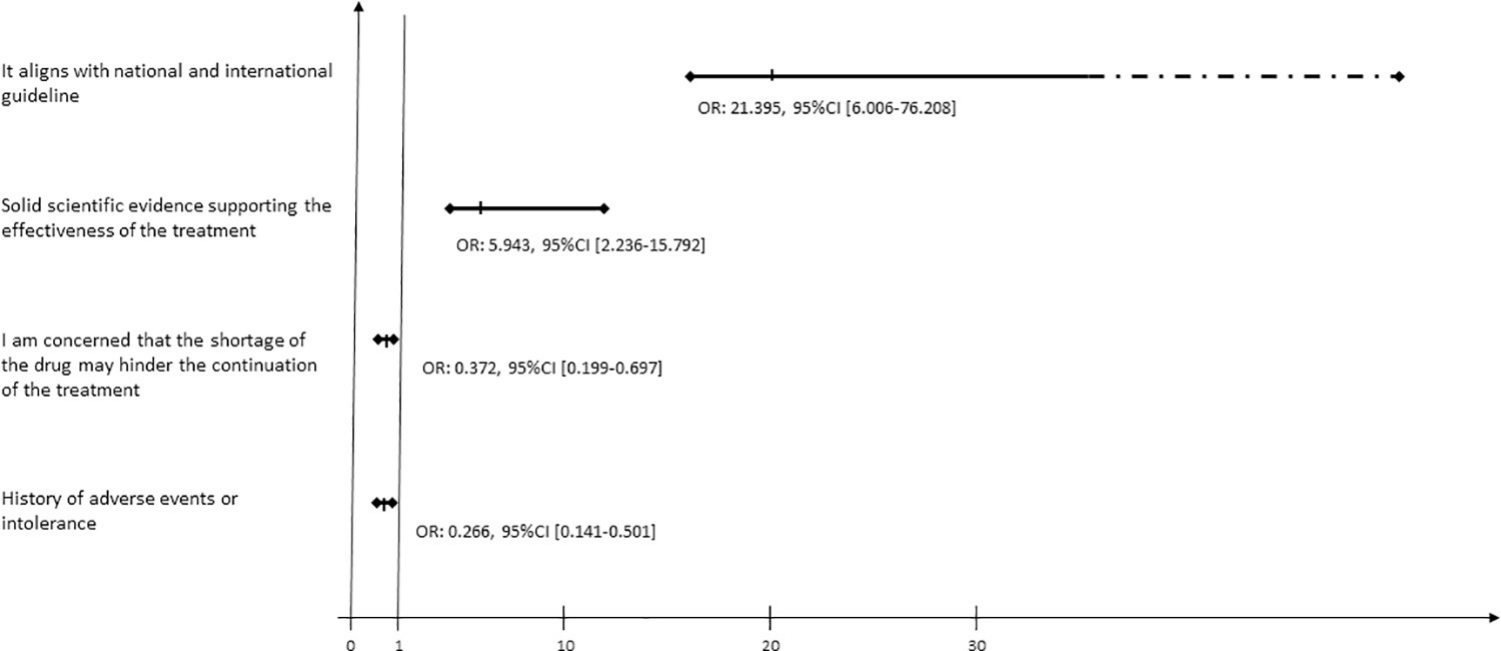
Motivations associated with FCHO.

In multivariate analysis, the following motivations were associated with FCHO: *It aligns with national and international guidelines* (OR: 21.395, 95%CI [6.006–76.208], p≤0.0001), *Solid scientific evidence supporting the effectiveness of the treatment* (OR: 5.943, 95%CI [2.236–15.792], p≤0.0001), *I am concerned that the shortage of the drug may hinder the continuation of the treatment* (OR: 0.372, 95%CI [0.199–0.697], p≤0.002) and *History of adverse events or intolerance* (OR: 0.266, 95%CI [0.141–0.501], p≤0.0001 (**Fig 2**).

## Discussion

The process of making treatment decisions is complex, dynamic, and pluralistic. It involves the interaction of multiple actors from the healthcare systems over time and should not be conceived as a single event between just the doctor and the patient [18]. Existing literature has primarily focused on cognitive processes and shared decision-making [18,22,25,26,36]. However, the field of affective science, which examines emotional elicitation, experiences, and emotion-driven behavior, is gaining recognition in clinical decision-making [28,37,38]. Various factors and actors interact to contribute to rheumatologists’ prescriptions [39–43]. However, most studies have focused on shared decision-making and RA diagnosis and have been performed mainly on patients from the USA and European countries [25]. It is important to note that the health system environment affects decision-making and varies across countries, which might be a limitation to currently fully comprehending the topic.

The study was conducted on a well-defined group of Mexican outpatients with various RMDs. The diagnoses were determined by the treating rheumatologists based on a complex process that extended beyond the application of classification criteria, as recommended [44]. The study took into account several motivations behind physician prescriptions, including the emotions of the physicians ("I am concerned that the shortage of the drug may hinder the continuation of the treatment…"), which have been shown to influence clinical decisions [7]. Three outcomes derived from physicians (detailed in the RMD outcomes form) were evaluated, and their performance had been previously tested in similar local patients [32]. In addition, PROMs were also assessed.

In our study, we found that FCHO was prescribed in the majority of medical appointments for outpatients with RMD. When FCHO was prescribed, it was associated with better outcomes as reported by physicians, compared to instances where SCHO was prescribed. We did not find any impact of FCHO on PROMs. It’s important to acknowledge that research on this topic in the context of RMDs is limited. However, existing literature on shared decision-making in rheumatology suggests that it can influence treatment choices [45–47]. Additionally, it can improve adherence to treatment plans and reduce medical costs [47–49]. These outcomes are all significant for physicians. Additionally, shared decision-making has been linked to improved PROMs such as patient satisfaction, knowledge, self-efficacy in healthcare settings, and trust in physicians [25]. While we recognize that the FCHO definition doesn’t equate to shared decision-making, it is aligned with the concept in that it represents a better option for influencing outcomes. We did not observe any impact on PROMs related to quality of life, physical function, and the patient-doctor relationship, which may be influenced by additional factors beyond the choice of treatment.

Second, we identified different motivations behind the types of prescriptions. For treating rheumatologists, the primary motivations for prescribing FCHO were often related to evidence-based medicine and personal experience. In contrast, SCHO prescriptions were more frequently motivated by concerns about past adverse events, intolerances, and drug shortages. Additionally, trainees in rheumatology were more likely to cite evidence-based medicine motivations, while certified rheumatologists considered social aspects such as patient benefit from gratuity and patient preferences. Aside from clinical indications, various factors can influence medication prescriptions, leading to individual variations that are not unexpected [39]. This phenomenon has been confirmed among trainees as well [50]. While scientific evidence of drug effects (both desired and undesired) has been considered a highly significant influence on prescriptions in some studies, there may be differing interpretations among specialists about the evidence [39]. In addition, other factors also play a crucial role in clinical decision-making, potentially explaining observed differences behind prescription motivations. Furthermore, significant variations have been observed in the implementation of evidence-based practice across professions, work units, and individuals [51]. It is generally acknowledged that physicians, especially those involved in research, are more likely to implement evidence-based practice [51,52]. An evidence-based climate and leadership, which are typically integrated into trainee academic environments, are seen as crucial in facilitating evidence-based practice [51,53,54]. Patient attitudes and preferences have also been found to influence prescription decisions among senior rheumatologists [39]. Lastly, long-standing relationships, particularly with certified rheumatologists, versus trainees, are known to contribute to increased patient involvement [39,40]. All the above published observations align with our results.

Third, motivations related to evidence-based medicine were associated with a higher likelihood of FCHO, while a history of adverse events/intolerance and concerns about drug shortages were associated with a lower likelihood. In rheumatology practice, treating rheumatologists usually applied FCHO, and studies have shown that the perceived personal ability to use evidence in practice is linked to evidence-based practice implementation [51]. However, subjective judgment and experience also play a significant role in prescription decisions [39,51], with some physicians more often relying on personal beliefs and experience, rather than on scientific evidence [40]. Various organizational factors also influence prescription decisions, including the impact of available resources [39]. The motivation "I am concerned that the shortage of the drug may hinder the continuation of the treatment for the necessary duration" reflects the subjective experience of emotion, which has been identified as a main theme in clinical decision-making [27]. Recognizing and mastering the emotional aspect of clinical decision-making has been suggested to be a critical element in improving patient safety [55,56]. Our results also align with Prosser et al.’s theoretical framework, which recognizes a combination of four types of knowledge (scientific, social, patient, and experiential) to influence drug prescription. Gaps in scientific knowledge can be filled through professional networks ("social knowledge") and previous experience ("experiential knowledge"), leading to variations in clinical practice.

The study has some limitations that need to be addressed. Firstly, this is a single-center study, and both patients and physicians had specific characteristics that might limit the generalizability of the results. The influence of the specific environment has been acknowledged and contributes to the preservation of treatment traditions within the clinic [39]. Secondly, all the patients included in the study had AC of the underlying RMD, although the severity of symptoms might influence decision-making [25]. Thirdly, we only investigated a limited number of reasons behind the choices made by rheumatologists. Fourthly, we used two questionnaires to assess physicians’ choices and RMD-related outcomes that have not undergone a formal validation process, although their performance was tested. Finally, patient inclusion in P-1 and Sub-P1 followed a systematic sampling method rather than randomization, which might have led to biased results.

## Conclusions

Patients with AC of their underlying RMD, whose treating rheumatologists prescribed their FCHO, had better physician-related outcomes than those who were prescribed SCHO, although PROMs were not impacted. There were differences in the motivations behind FCHO and SCHO; treating rheumatologists’ FCHO were often based on evidence-based medicine motivations and personal experience, while SCHO more frequently stemmed from concerns about potential drug shortages, previous adverse events or intolerance, and drug shortages. Scientific evidence-based motivations, rheumatologists concern of medication shortage, and patient-related motivations were associated with FCHO.

## Supporting information

S1 Checklist STROBE statement—Checklist of items that should be included in reports of *cohort studies*. (DOC)

S1 Appendix Treatment choice instrument. (DOCX)

S2 Appendix RMD outcomes questionnaire. (DOCX)

S1 Table N° (%) of patients with at least one visit to the outpatient clinic with the 10 most frequent diagnoses specified (January to December 2023). (DOCX)

S2 Table Categories related to the RMD clinical status and the corresponding pre-specified criteria. (DOCX)

S3 Table Comparison of physicians´ motivations behind FCHO and SCHO, in SubP-1. (DOCX)
