# Supplementary material for: The choice of treatment and the motivations behind it impact clinical outcomes among patients with adequate control of their rheumatic disease: A real-life study
Source: PLoS One. 2024 Dec 12;19(12):e0315478. doi: 10.1371/journal.pone.0315478 (PMC11637349; doi:10.1371/journal.pone.0315478)
Supplement: S1 Appendix — (DOCX) [file pone.0315478.s002.docx]

**Appendix 1. Treatment choice instrument.**

Considering this particular patient, the treatment corresponds to:

-------- Your first option

-------- Your second option

Please select all the motivations behind your choice of RMD-related treatment, considering the specific clinic case and the patient´s context.

| **Physician-related** | | | |
| --- | --- | --- | --- |
|  | Yes | No |  |
| It aligns with national and international guidelines. |  |  |  |
| There is solid scientific evidence supporting the effectiveness of the treatment. |  |  |  |
| I have personal experience with that treatment. |  |  |  |
| I am concerned that the shortage of the drug may hinder the continuation of the treatment for the necessary duration. |  |  | If yes, please specify the drug. |
| Other reasons. |  |  | Specify. |
| **Patient-related** | | | |
|  | Yes | No |  |
| Socio-demographics (age, education level, etc...). |  |  |  |
| Relevant comorbidities. |  |  | The patient does not have relevant comorbidities. |
| History of adverse events or intolerance. |  |  | The patient has no history of adverse events or intolerance. |
| Economic motivations: the patient can´t afford the treatment. |  |  | This does not apply because the patient has access to free medications. |
| Patients´ preference. |  |  |  |
| Other reasons. |  |  |  |
| **Health-care system related** | | | |
|  | Yes | No |  |
| Local shortage |  |  | The patient does not have access to free medications.  If yes, specify the drug. |
| National shortage |  |  | If yes, specify the drug. |
| Patient benefits from local gratuity |  |  |  |
| Patient benefits from social security gratuity |  |  |  |
| Other reasons. |  |  | Specify. |
