# Supplementary material for: The choice of treatment and the motivations behind it impact clinical outcomes among patients with adequate control of their rheumatic disease: A real-life study
Source: PLoS One. 2024 Dec 12;19(12):e0315478. doi: 10.1371/journal.pone.0315478 (PMC11637349; doi:10.1371/journal.pone.0315478)
Supplement: S2 Appendix — (DOCX) [file pone.0315478.s003.docx]

**Appendix 2. RMD outcomes questionnaire.**

1. Considering the patient's clinical expression, how will you define the current level of disease activity?

| Without disease activity |  |
| --- | --- |
| Substantial disease activity (low, moderate or severe) |  |

2.- How would you rate the current control of the rheumatic disease?

| Adequate control |  |
| --- | --- |
| Inadequate control |  |

3.- During the visit, did you suggest or make any changes to the treatment for rheumatic disease?

| No changes. |  |
| --- | --- |
| Treatment was modified because of RMD improvement. |  |
| Treatment was modified because of RMD deterioration/insufficient response. |  |
| Treatment was modified due to drug toxicity. |  |
| Treatment was modified because of non-adherence. |  |
