# Supplementary material for: The choice of treatment and the motivations behind it impact clinical outcomes among patients with adequate control of their rheumatic disease: A real-life study
Source: PLoS One. 2024 Dec 12;19(12):e0315478. doi: 10.1371/journal.pone.0315478 (PMC11637349; doi:10.1371/journal.pone.0315478)
Supplement: S1 Table — (DOCX) [file pone.0315478.s004.docx]

**Supplementary Table 1. N° (%) of patients with at least one visit to the outpatient clinic with the 10 most frequent diagnoses specified (January to December 2023).**

| **RMD diagnoses¹** | **Nº (%) of patients**  **n=6852** |
| --- | --- |
| SLE | 2251 (32.9) |
| RA | 2116 (30.9) |
| Sclerodermia | 357 (5.2)) |
| Primary Sjögren Syndrome (PSS) | 282 (4.1) |
| Inflammatory Miopathies (IM) | 266 (3.9) |
| Systemic Vasculitis (SV) | 237 (3.5) |
| Primary Anti-Phospholipid Syndrome (PAPS) | 199 (2.9) |
| Spondyloarthritis (SA) | 197 (2.9) |
| Osteoarthritis | 98 (1.4) |
| Mixed Connective Tissue Disease (MCTD) | 83 (1.2) |
| Other diagnosis | 632 (9.2) |
| Without a defined RMD diagnosis | 134 (2.0) |

*¹Diagnosed based on the attending rheumatologist criteria. SLE=Systemic Lupus Erythematosus. RA=Rheumatoid Arthritis.*
