# Supplementary material for: The choice of treatment and the motivations behind it impact clinical outcomes among patients with adequate control of their rheumatic disease: A real-life study
Source: PLoS One. 2024 Dec 12;19(12):e0315478. doi: 10.1371/journal.pone.0315478 (PMC11637349; doi:10.1371/journal.pone.0315478)
Supplement: S2 Table — (DOCX) [file pone.0315478.s005.docx]

**Supplementary Table 2. Categories related to the RMD clinical status and the corresponding pre-specified criteria.**

| **Categories** | **Sub-categories** | **Pre-specified criteria** |
| --- | --- | --- |
| **The level of disease activity (at the current evaluation** | Without disease activity. | No symptoms AND no clinical findings AND relevant serological markers within normal values. |
|  | Substantial disease activity level. | Two out of 3 of the following: Symptoms, clinical findings, and relevant serological markers. |
| **RMD control** | Adequate control of the rheumatic disease. | Symptoms (if any) AND clinical findings (if any) AND serological markers within an acceptable target AND that do not require treatment adjustment. |
|  | Inadequate/Insufficient control of the rheumatic disease. | Two out of 3 of the following: Symptoms, clinical findings, relevant serological markers, out of target, and that require treatment adjustment. |
| **Suggested treatment modifications** | No changes. | No changes |
|  | Treatment was modified because of RMD improvement. | Drug(s) discontinuation/dose reduced/prolonged interval administration or a change to a scheme lesser intensive. |
|  | Treatment was modified because of RMD deterioration/insufficient response. | Drug(s) initiation, addition, increased dose.  Change to a more intensive scheme.  Intra-articular injection. |
|  | Treatment was modified due to drug toxicity. | Any modification for the reason specified. |
|  | Treatment was modified because of non-adherence. | Non-adherence could be related to medication shortage, economic reasons, etc… |
