# Supplementary material for: The choice of treatment and the motivations behind it impact clinical outcomes among patients with adequate control of their rheumatic disease: A real-life study
Source: PLoS One. 2024 Dec 12;19(12):e0315478. doi: 10.1371/journal.pone.0315478 (PMC11637349; doi:10.1371/journal.pone.0315478)
Supplement: S3 Table — (DOCX) [file pone.0315478.s006.docx]

**Supplementary Table 3. Comparison of physicians´ motivations behind FCHO and SCHO, in SubP-1.**

|  | **FCHO**  **N=269** | **SCHO**  **N=23** | **p** |
| --- | --- | --- | --- |
| **Physician-related** | | | |
| It aligns with national and international guidelines. | 266 (98.9) | 17 (73.9) | ≤0.0001 |
| There is solid scientific evidence supporting the effectiveness of the treatment. | 264 (98.1) | 20 (87) | 0.019 |
| I have personal experience with that treatment. | 263 (97.8) | 20 (87) | 0.026 |
| I am concerned that the shortage of the drug may hinder the continuation of the treatment for the necessary duration. | 51 (19) | 12 (52.2) | 0.001 |
| Other reasons¹. | 10 (3.7) | 3 (13) | 0.073 |
| **Patient-related** | | | |
| Socio-demographics (age, education level, etc...). | 121 (45) | 12 (52.2) | 0.521 |
| Relevant comorbidities. | 107 (39.8) | 11 (47.8) | 0.509 |
| History of adverse events or intolerance. | 42 (15.6) | 8 (34.8) | 0.037 |
| Economic motivations: the patient can´t afford the treatment. | 57 (21.2) | 3 (13) | 0.433 |
| Patients´ preference. | 105 (39) | 7 (30.4) | 0.506 |
| Other reasons². | 11 (4.1) | 1 (4.3) | 1 |
| **Health-care system related** | | | |
| Local shortage | 34 (12.6) | 8 (34.8) | 0.009 |
| National shortage | 14 (5.2) | 2 (8.7) | 0.365 |
| Patient benefits from local gratuity | 159 (59.1) | 13 (56.5) | 0.828 |
| Patient benefits from social security gratuity | 59 (21.9) | 4 (17.4) | 0.794 |
| Other reasons³. | 2 (0.7) | 1 (4.3) | 0.219 |

*Data presents the number (%) of physicians who selected that motivation. ¹No reasons were specified in 65.2% of the treatment choice questionnaires with the “Other reasons” option selected. ²No reasons were specified in 57.5% of the treatment choice questionnaires with the “Other reasons” option selected, while pregnancy/pregnancy planning/breastfeeding were referred in 19.2%. ³No reasons were specified in 94.5% of the treatment choice questionnaires with the Other reasons” option selected.*
